# Association between the Multidimensional Poverty Measure and COVID-19 mortality in Colombia

**DOI:** 10.1093/eurpub/ckac130.088

**Published:** 2022-10-25

**Authors:** JP Perez Bedoya, OI Mendoza Cardozo, CA Pérez Aguirre, LM Ruiz Galvis, JP Sánchez Escudero, J Cardona Jimenez, BA Rodríguez Rey, NC Barengo, PA Diaz Valencia

**Affiliations:** 1 National School of Public Health, University of Antiquia, Medellín, Colombia; 2 Statistics Institute, National University of Colombia, Medellín, Colombia; 3 Physics Institute, University of Antiquia, Medellín, Colombia; 4 Herbert Wertheim College, Florida International University, Miami, Colombia

## Abstract

**Background:**

Different socioeconomic aspects have been related to mortality from COVID-19. For this reason, the objective of this study was to analyze the association between the Multidimensional Poverty Measure at the municipal level (MPM) and the clinical outcome of mortality in the resident population of Colombia with a diagnosis of COVID-19.

**Methods:**

Observational, non-concurrent cohort study of confirmed cases of COVID-19 reported in Colombia by August 2021. The main outcome variable was mortality from COVID-19, and the main exposure variable was MPM. The covariates included in the analysis were patient's sex, age, and municipality of residence. Unadjusted and adjusted logistic models were used using balanced random samples of deaths and recovered patients, calculating odds ratios (OR) and 95% confidence interval ranges (CI).

**Results:**

In total, 4,194,538 cases of COVID-19 were included in the analysis, of which approximately 3% died. According to the adjusted multivariate analysis, it was found that patients who live in municipalities with an MPM between 20 to 40%, 41 to 60%, 61 to 80% and more than 80% had an OR of 1.6 (95% CI 1.4 to 1.8), 1.6 (95% CI 1.3 to 1.9), 1.7 (95% CI 1.2 to 2.5), and 2.2 (95% CI 0.7 to 7.8), respectively, for mortality from COVID-19 compared with an MPM of less than 20%. When analyzing the data according to sex for the MPM from 20 to 40%, 41 to 60%, 61 to 80% and more than 80%, an OR for women of 1.7 (95% CI 1.5 to 2.0), 1.8 (95% CI % 1.5 to 2.1), 1.9 (95% CI 1.3 to 2.6) and 2.8 (CI 0.9 to 10.1) respectively. For men an OR of 1.5 (95% CI 1.3 to 1.7), 1.4 (95% CI 1.2 to 1.7), 1.6 (95% CI 1.1 to 2.3) and 1.9 (95% CI 0.6 to 6.0) respectively compared to a MPM less than 20%.

**Conclusions:**

The risk of mortality from COVID-19 in Colombia is increased in populations with higher MPM. Social determinants of health have an important effect on the outcomes of COVID-19.

**Key messages:**

- Only a few studies exist in South America on social determinants in health and COVID-19. This study provides evidence to understand the impact of poverty measures on mortality in COVID-19 patients.
- Multidimensional poverty measure at the municipal level was associated with increased COVID-19 mortality in a large cohort of patients from the Colombian population.

